# Coenzyme Q_10_ Status as a Determinant of Muscular Strength in Two Independent Cohorts

**DOI:** 10.1371/journal.pone.0167124

**Published:** 2016-12-01

**Authors:** Alexandra Fischer, Simone Onur, Petra Niklowitz, Thomas Menke, Matthias Laudes, Gerald Rimbach, Frank Döring

**Affiliations:** 1 Department of Molecular Prevention, Institute of Human Nutrition and Food Science, Christian-Albrechts-University of Kiel, Kiel, Germany; 2 Children’s Hospital of Datteln, Witten/Herdecke University, Datteln, Germany; 3 Department of Internal Medicine, University Hospital Schleswig-Holstein, Campus Kiel, Kiel, Germany; 4 Department of Food Science, Institute of Human Nutrition and Food Science, Christian-Albrechts-University of Kiel, Kiel, Germany; Leibniz-Institut fur Pflanzengenetik und Kulturpflanzenforschung Gatersleben, GERMANY

## Abstract

Aging is associated with sarcopenia, which is a loss of skeletal muscle mass and function. Coenzyme Q_10_ (CoQ_10_) is involved in several important functions that are related to bioenergetics and protection against oxidative damage; however, the role of CoQ_10_ as a determinant of muscular strength is not well documented. The aim of the present study was to evaluate the determinants of muscular strength by examining hand grip force in relation to CoQ_10_ status, gender, age and body mass index (BMI) in two independent cohorts (n = 334, n = 967). Furthermore, peak flow as a function of respiratory muscle force was assessed. Spearman’s correlation revealed a significant positive association between CoQ_10_/cholesterol level and hand grip in the basic study population (p<0.01) as well as in the validation population (p<0.001). In the latter, we also found a negative correlation with the CoQ_10_ redox state (p<0.01), which represents a lower percentage of the reduced form of CoQ_10_ (ubiquinol) in subjects who exhibit a lower muscular strength. Furthermore, the age of the subjects showed a negative correlation with hand grip (p<0.001), whereas BMI was positively correlated with hand grip (p<0.01), although only in the normal weight subgroup (BMI <25 kg/m^2^). Analysis of the covariance (ANCOVA) with hand grip as the dependent variable revealed CoQ_10_/cholesterol as a determinant of muscular strength and gender as the strongest effector of hand grip. In conclusion, our data suggest that both a low CoQ_10_/cholesterol level and a low percentage of the reduced form of CoQ_10_ could be an indicator of an increased risk of sarcopenia in humans due to their negative associations to upper body muscle strength, peak flow and muscle mass.

## Introduction

Sarcopenia is a common problem in the Western world. It is characterized by progressive and generalized loss of skeletal muscle mass and strength with the risk of adverse outcomes, such as physical disability, poor quality of life and death [1,2]. Furthermore, impairment in skeletal muscle function is age-related and associated with a decrease in fiber number and an increase in extramyocyte space [3]. The fiber cross-sectional area and amount of connective tissue undergo significant age-related changes [4].

Coenzyme Q_10_ (CoQ_10_) plays a crucial role in mitochondrial bioenergetics, including ATP production [5,6]. Furthermore, CoQ_10_ could act as an antioxidant, preventing oxidative damage of lipids, proteins and DNA [7,8]. Moreover, CoQ_10_ has been identified as a modulator of gene expression [9, 10] and is necessary for the function of uncoupling proteins [11] and pyrimidine biosynthesis [12]. The percentage of the oxidized form of CoQ increases with age, indicating a decreased anti-oxidative capacity of aged individuals [13]. On the other hand, we have found that the phenotypic characteristics of senescence in SAMP1 mice can be partly counteracted by supplementation with the reduced form of CoQ_10_ [14]. Previous reports have documented different contributors to sarcopenia (including TNF-α-dependent apoptotic signaling), which could be potential targets for CoQ_10_. Hence, pro-apoptotic responses to TNF-α are mediated by activation of the plasma membrane neutral sphingomyelin phosphodiesterase (SMPD2) [15]. Navas and coworkers have previously demonstrated that SMPD activity is regulated by CoQ through noncompetitive inhibition of the enzyme [16,17]. Dietary supplementation with CoQ abolished age-related increases of SMPD activity in the rat liver plasma membrane [18]. Moreover, it was shown that a higher expression of muscle peroxisome proliferator-activated receptor γ coactivator 1α (PGC-1α), a major factor that controls mitochondrial biogenesis and respiration, protects from sarcopenia during aging [19] and that PGC-1α expression was increased in SAMP1 mice by dietary supplementation of reduced CoQ_10_ (ubiquinol) [20].

In the present study, we aimed to investigate the determinants of muscular strength with a particular focus on CoQ_10_ in two independent cohorts that compromise a total of n = 1301 subjects. The European Working Group on Sarcopenia in Older People (EWGSOP) has recommended hand grip strength as a robust and simple measure of muscle strength [21]. Furthermore, reduced grip strength has been associated with an increased risk of all-cause and cardiovascular mortality and is even a stronger predictor than systolic blood pressure [22,23]. Thus, in our study, the values for hand grip were correlated with the CoQ_10_ status, age, BMI and peak expiratory flow (PEF) as a measure of respiratory muscle function, which could also be affected in age-associated alterations in skeletal muscles [24]. Accordingly, the EWGSOP identified PEF as an alternative measure of muscle strength. To evaluate the influence of the variables that predict the outcome of muscular strength independently, analysis of covariance (ANCOVA) was performed with hand grip as the dependent variable. Because cholesterol is the main transport vehicle for CoQ_10_ in serum [7,25] and cholesterol and CoQ_10_ share parts of a common synthetic pathway [26], the plasma CoQ_10_/cholesterol ratio was used in ANCOVA. Additionally in the basic study population, the muscle mass, creatinine content and creatine kinase activity were determined.

## Materials and Methods

### Participants and Ethical Statement

The basic study population consisted of 334 apparent healthy blood donors and is part of the PopGen control cohort [27,28]. The validation population (n = 967) is part of the FoCus cohort [29]. The participants in this European study collective were recruited in cooperation with the University Hospital Schleswig-Holstein (UKSH), Kiel, Germany as healthy blood donors or in the adiposity ambulance of UKSH. Both populations have been described recently [30] and raw data of participants are given in S1 Table

The present study was conducted according to the guidelines laid down in the Declaration of Helsinki, and all of the procedures that involve human subjects were approved by the ethics committee of the Medical Faculty of the Christian-Albrechts University of Kiel. All participants gave their written informed consent before participation.

### CoQ_10_ and blood parameters

Analysis of ubiquinol-10 and ubiquinone-10 was based on the method of HPLC with electrochemical detection after hexane extraction and has been described before [31]. Ubiquinol-9 and ubiquinone-9 were used as internal standards.

Blood samples were taken from every participant after an overnight fast. Serum concentrations of total cholesterol were measured by an enzymatic colorimetric assay (Hitachi Modular; Roche). Creatinine and creatine kinase were analyzed by standard clinical chemistry.

### Physiological measurements

Standing height and weight were measured in light clothing without shoes. Body mass index (BMI) was calculated by dividing the weight (kg) by the square of the height (m^2^). Total plasma creatinine values were used to calculate the muscle mass in the basic study population [32,33]. Beforehand, the plasma volume was determined by using the equation by Sprenger et al. [34], which considers the height, weight and hematocrit of the participants.

Upper muscular strength (hand grip strength) was characterized with a digital dynamometer (MAP 80K1, Kern, Balingen, Germany). The participants maintained the standard bipedal position during the entire test with the arm in complete extension. Each participant performed the test three times with each hand. The highest value was chosen as the test score for each hand (dominant and non-dominant hand), and an average score for both hands was computed as the bimanual hand grip score according to [8]. Peak flow was measured by having the person exhale as forcefully as possible through the peak flow meter (Mini Wright Standard) after a maximum inspiration.

### Statistical analysis

Statistical analysis was performed using IBM SPSS 23.0 software (Armonk, New York, USA) and GraphPad Prism 4.0 package (La Jolla, California, USA). The data are expressed as the mean ± SD, and statistical significance was set at p<0.05. After testing for normality (Kolmogorov-Smirnov), significant differences between sexes were analyzed by a two-sided, unpaired Student’s t-test in the case of normally distributed parameters or by the Mann-Whitney-U-Test in the case of non-normally distributed parameters. Correlation analysis (Spearman’s or Pearson’s correlation, dependent on the distributions of the parameters) was conducted for all of the measured variables in both of the study populations and separately for both genders. Analysis of covariance (ANCOVA) was performed on both cohorts, with “hand grip” as the dependent variable. Gender was taken as an independent factor and BMI, age and CoQ_10_/cholesterol as independent covariates. Prior to ANCOVA, the different terms were controlled for the significance of interaction.

## Results

### Characterization of the basic study population

The age, body composition (weight, height, BMI, muscle mass), and biochemical profile, including the CoQ_10_ status and functional status (hand grip, peak flow), of the study population, separated by gender, are shown in Table 1. The total study population (n = 178 males, n = 156 females) had a mean age of 40.4 ± 11.2 years (age range 19–61 years), and all of the parameters evaluated were within the normal range, including BMI, total CoQ_10_, CoQ_10_ redox state (% oxidized CoQ_10_ in total), peak flow and hand grip. As expected, men had a significantly higher body weight, height, BMI, muscle mass, peak flow and hand grip compared to females. With regard to the biochemical profile, men also showed higher serum levels of total CoQ_10_, CoQ_10_/cholesterol, creatine kinase and creatinine.

**Table 1:** Characterization of the basic study population (*n* = 334).

	Males (*n* = 178)		Females (*n* = 156)		Total (*n* = 334)	
Parameter		r *vs*. hand grip^a^		r *vs*. hand grip^a^		r *vs*. hand grip^a^
**Age (years)**	41.0 ± 10.9	0.008	39.8 ± 11.5	−0.258	40.4 ± 11.2	−0.015
		p = 0.918		**p<0.01**		p = 0.780
**Weight (kg)**	87.6 ± 14.7	0.245	73.1 ± 14.7***	0.271	80.8 ± 16.4	0.525
		**p<0.01**		**p<0.01**		**p<0.001**
**Height (cm)**	182.1 ± 7.3	0.207	168.9 ± 6.75***	0.242	175.9 ± 9.6	0.672
		**p<0.01**		**p<0.01**		**p<0.001**
**Muscle mass**^**b**^ **(kg)**	32.6 ± 5.15	0.172	19.8 ± 3.92***	0.306	26.7 ± 7.89	0.744
		**p<0.05**		**p<0.001**		**p<0.001**
**Peak flow (L/min)**	525.3 ± 98.5	0.316	388.2 ± 55.1***	0.274	461.3 ± 106.2	0.525
		**p<0.001**		**p<0.01**		**p<0.001**
**BMI (kg/m**^**2**^**)**	26.4 ± 4.08	0.138	25.6 ±4.79**	0.175	26.0 ± 4.4	0.212
		p = 0.067		**p<0.05**		**p<0.001**
**CoQ**_**10**_ **(μg/mL)**	0.978 ± 0.37	0.004	0.872 ± 0.29**	-0.099	0.929 ± 0.34	0.103
		p = 0.955		p = 0.217		p = 0.061
**CoQ**_**10**_ **redox (%)**	12.1 ± 2.53	0.073	12.4 ± 2.20	−0.067	12.3 ± 2.4	−0.066
		p = 0.333		p = 0.407		p = 0.232
**CoQ**_**10**_**/cholesterol (μmol/mol)**	193.7 ± 53.4	0.004	177.3 ± 54.1**	-0.025	186.0 ± 54.4	0.154
		p = 956		p = 0.756		**p<0.01**
**Cholesterol (mmol/L)**	5.01 ± 1.03	-0.015	4.91 ± 0.77	-0.148	4.96 ± 0.917	0.013
		p = 0.844		p = 0.066		p = 0.812
**Creatine kinase (U/L)**	166.1 ± 94.1	0.013	106.7 ±62.7***	0.018	138.4 ± 86.2	0.365
		p = 0.865		p = 0.827		**p<0.001**
**Creatinine (mg/dL)**	0.904 ± 0.12	0.039	0.713 ± 0.11***	0.063	0.815 ±0.151	0.570
		p = 0.604		p = 0.436		**p<0.001**
**Hand grip**_**Dominant hand**_ **(kg)**	52.4 ± 8.63		32.5 ± 5.88***		43.1 ± 12.4	

Data are presented as the mean ± SD. BMI: body mass index; CoQ_10_: total Coenzyme Q_10_; CoQ_10_ redox: % oxidized CoQ_10_ in total.

^a^r = Spearman’s correlation coefficient between the hand grip and the evaluated parameter in the total study population. In the case of a normal distribution (muscle mass, cholesterol), Pearson’s correlation was applied.

^b^Muscle mass was calculated according to [32–34].

*p<0.05

**p<0.01

***p<0.001 significant differences between the sexes, evaluated by the Mann-Whitney-U-Test after testing for normality (Kolmogorov-Smirnov). In case of a normal distribution (muscle mass; cholesterol), Student’s t-test was applied.

**Fig 1:**
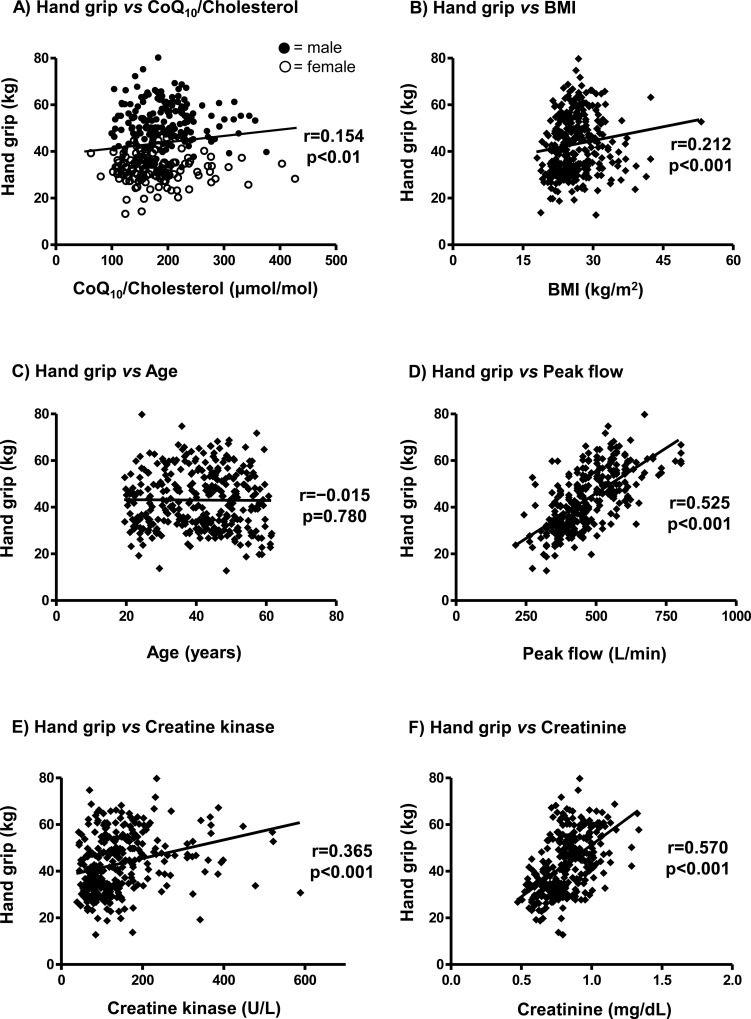
Scatterplots of correlations between hand grip and CoQ_10_/cholesterol, body mass index, age, peak flow, creatine kinase and creatinine in the basic study population (n = 334). Spearman’s correlation analysis revealed a significant relationship (p<0.01) between hand grip and CoQ_10_/cholesterol (A), BMI (B), peak flow (C), creatine kinase (E) and creatinine (F), whereas the correlation between hand grip and age (C) was statistically not significant. Spearman’s correlation coefficient (r), p-values and regression lines are given. CoQ_10_: Coenzyme Q_10_; BMI: body mass index.

We hypothesized that the selected parameters (as age, BMI and CoQ_10_ status) are determinants of hand grip, a valid proxy of upper-body muscular strength (Table 1, Fig 1). Correlation analysis of the total study population to hand grip revealed significant positive associations between hand grip and CoQ_10_/cholesterol (r = 0.154, p<0.01, Fig 1A), BMI (r = 0.212, p<0.001, Fig 1B), peak flow (r = 0.525, p<0.001, Fig 1D), creatine kinase (r = 0.365, p<0.001, Fig 1E) and creatinine (r = 0.570, p<0.001, Fig 1F). The highest correlation coefficient was evident between hand grip and muscle mass (r = 0.744, p<0.001). No significant association was evident between hand grip and age (r = −0.015, p = 0.780, Fig 1C), total CoQ_10_ (r = 0.103, p = 0.061), CoQ_10_ redox (r = -0.066, p = 0.232) and total cholesterol (r = 0.013, p = 0.812). Except for the parameters BMI (r = 0.138, p = 0.067) and age (r = 0.008, p = 0.918), which were only significantly correlated to hand grip in females, similar outcomes were found when both genders were correlated separately.

### Characterization of the validation population

To further evaluate the determinants of muscular strength, we validated our results derived from the basic study population on a second, independent cohort. The characteristics of this validation population are given in Table 2. This population consists of 341 males and 626 females for a total of 967 subjects; they exhibited a mean age of 52.6 ± 14.1 years (age range: 20–85 years). Thus, this population was, on average, 10 years older than the basic study population. Furthermore, among the validation population, 468 obese study subjects (BMI >30, mean BMI: 40.8 ± 8.52 kg/m^2^) were incorporated to provide a broader BMI range. In total, the population showed a mean BMI of 32.2 ± 10.4 kg/m^2^, which is considered to be obese. The remaining parameters, however, were within the normal range, including the total CoQ_10_,CoQ_10_ redox state (oxidized CoQ_10_ in total) and hand grip. Similarly, males had a significantly higher body weight, height, total CoQ_10_ levels and hand grip and were on average 6 years older than females.

**Table 2:** Characterization of the validation population (*n* = 967), including 658 overweight/obese study subjects.

	Males (*n* = 341)		Females (*n* = 626)		Total (*n* = 967)	
Parameter		r *vs*. hand grip^a^		r *vs*. hand grip^a^		r *vs*. hand grip^a^
**Age (years)**	56.1 ± 13.5	-0.427	50.6 ± 14.5***	-0.416	52.6 ± 14.1	-0.137
		**p<0.001**		**p<0.001**		**p<0.001**
**Weight (kg)**	102.3 ± 33.2	0.195	92.6 ± 32.1***	0.230	96.0 ± 32.8	0.270
		**p<0.001**		**p<0.001**		**p<0.001**
**Height (cm)**	180.5 ± 8.5	0.331	168.0 ± 6.7***	0.434	172.4 ± 9.5	0.695
		**p<0.001**		**p<0.001**		**p<0.001**
**BMI (kg/m**^**2**^**)**	31.3 ± 9.0	0.115	32.8 ± 11.0	0.131	32.2 ± 10.4	0.056
		**p<0.05**		**p<0.01**		p = 0.082
**BMI <25 (normal)**	23.2 ± 1.59	0.301	22.1 ± 2.07	0.170	22.4 ± 2.01	0.170
	(*n* = 88)	**p<0.01**	(*n* = 221)	**p<0.01**	(*n* = 309)	**p<0.01**
**BMI 25–30 (overweight)**	27.3 ± 1.13	−0.144	27.3 ± 1.19	−0.110	27.3 ± 1.16	−0.110
	(*n* = 112)	p = 0.131	(*n* = 78)	p = 0.131	(*n* = 190)	p = 0.131
**BMI >30 (obese)**	39.4 ± 8.68	-0.044	41.4 ± 8.39	0.002	40.8 ± 8.52	0.002
	(*n* = 141)	p = 0.601	(*n* = 327)	p = 0.973	(*n* = 468)	p = 0.973
**CoQ**_**10**_ **(μg/mL)**	0.852 ± 0.30	-0.071	0.801 ± 0.28**	-0.120	0.819 ± 0.29	-0.002
		p = 0.193		**p<0.01**		p = 0.946
**CoQ**_**10**_ **redox (%)**	13.4 ± 2.31	-0.035	13.6 ± 2.08*	-0.059	13.5 ± 2.17	-0.082
		p = 0.525		p = 0.143		**p<0.01**
**CoQ**_**10**_**/cholesterol (μmol/mol)**	193.1 ± 56.0	-0.088	168.8 ± 49.3***	-0.029	177.3 ± 53.0	0.131
		p = 0.104		p = 0.476		**p<0.001**
**Cholesterol (mmol/L)**	4.41 ± 0.887	0.046	4.76 ± 0.989	-0.207	4.63 ± 0.968	-0.185
		p = 0.393		**p<0.001**		**p<0.001**
**Hand grip**_**Dominant hand**_ **(kg)**	42.5 ± 9.4		25.0 ± 6.4***		31.2 ± 11.3	

Data are presented as the mean ± SD. BMI: body mass index; CoQ_10_: total Coenzyme Q_10_; CoQ_10_ redox: % oxidized CoQ_10_ in total.

^a^r = Spearman’s correlation coefficient between the hand grip and the evaluated parameter in the total study population. In the case of a normal distribution (cholesterol), Pearson’s correlation was applied.

*p<0.05

**p<0.01

***p<0.001 significant differences between the sexes, evaluated by the Mann-Whitney U-Test after testing for normality (Kolmogorov-Smirnov). In the case of a normal distribution (hand grip; cholesterol), Student’s t-test was applied.

**Fig 2:**
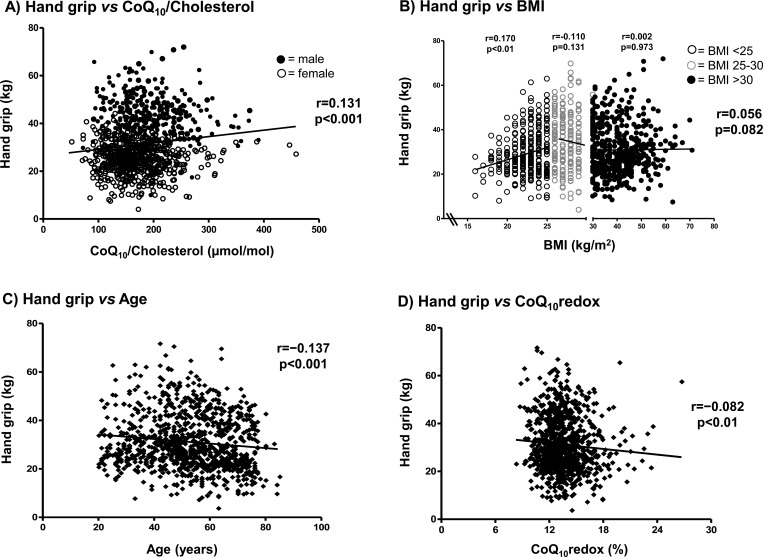
Scatterplots of correlations between hand grip and CoQ_10_/cholesterol ratio, body mass index, age and CoQ10 redox in the validation population (n = 967). Spearman’s correlation analysis revealed a significant relationship (p<0.01) between hand grip and CoQ_10_/cholesterol (A), normal BMI (<25 kg/m^2^, B), age (C) and CoQ_10_ redox (D). The correlations between hand grip and overweight (BMI 25–30 kg/m^2^) and obese subjects (BMI >30) were statistically not significant. Spearman’s correlation coefficient (r), p-values and regression lines are given. CoQ_10_: Coenzyme Q_10_; CoQ_10_ redox: % oxidized coenzyme Q_10_ in total; BMI: body mass index.

To substantiate our results in the basic study population, we correlated the parameters that were available with the values of the hand grip measurements (Table 2, Fig 2). Accordingly, correlation analysis revealed a significant positive association between hand grip and the CoQ_10_/cholesterol ratio (r = 0.131, p<0.001, Fig 2A) and a negative significant association to cholesterol itself (r = -0.185, p<0.001). Hand grip correlated with BMI in the total validation population and revealed no significant relationship (r = 0.056, p = 0.082). However, when we examined the BMI classes separately (Fig 2B), we found a significant correlation in subjects who exhibited a normal BMI (<25 kg/m^2^, r = 0.170, p<0.01), but not in overweight subjects (BMI 25–30 kg/m^2^, r = -0.110, p = 0.131) or obese subjects (BMI >30, r = 0.002, p<0.973). Furthermore, hand grip was negatively correlated with age (r = -0.137, p<0.001, Fig 2C) and the CoQ_10_ redox state (r = -0.082, p<0.01, Fig 2D), which represents a lower percentage of the reduced form of CoQ_10_ (ubiquinol) in subjects who exhibited lower muscular strength. In the validation population, similar outcomes were found when males and females were correlated separately with hand grip, with the exception of total CoQ_10_ (r = -0.071, p = 0.193) and cholesterol (r = -0.046, p = 0.393), which were only significantly correlated in females.

### CoQ_10_ status and muscular strength stratified according to Gender, Age and BMI

To independently evaluate the influence of the variables that predict the outcome of the upper body muscular strength (hand grip) in the basic study population, analysis of covariance (ANCOVA) was performed with hand grip as the dependent variable. In a model, including the gender of the study subjects as the independent factor and CoQ_10_/cholesterol, age and BMI as the independent covariates, gender was found to be the main effector (p<0.001, partial η^2^ = 0.636, data not shown). Next, we analyzed the remaining variables independent of gender, which revealed a significant effect on hand grip according to BMI (p<0.05, η^2^ = 0.017), whereas CoQ_10_/cholesterol (p = 0.099) and age (p = 0.563) were not significant predictors of hand grip in this study population (Table 3A).

**Table 3:** Analysis of covariance (ANCOVA) between hand grip, CoQ_10_/cholesterol ratio, age and BMI in A) the basic study population (n = 334) and B) the validation population (n = 967), including 658 overweight/obese subjects.

Source	Type III sum of Squares	df	*F*	Significance	Partial η^2^
**A) Basic study population**					
**Corrected Model**	1,477^**a**^	3	3.24	0.022	0.029
**Intercept**	6491	1	42.7	<0.001	0.115
**CoQ**_**10**_**/cholesterol**	416	1	2.74	0.099	0.008
**Age**	50.9	1	0.336	0.563	0.001
**BMI**	872	1	5.74	0.017	0.017
**B) Validation population**					
**Corrected Model**	4,049^**b**^	3	10.8	<0.001	0.033
**Intercept**	28,617	1	230.1	<0.001	0.193
**CoQ**_**10**_**/cholesterol**	2,266	1	18.2	<0.001	0.019
**Age**	2164	1	17.4	<0.001	0.018
**BMI**	2.13	1	0.017	0.896	<0.001

**^a^R^2^ = _0.033_ (Adjusted R^2^ = _0.030_),** dependent variable: hand grip, independent covariate: age, CoQ_10_/cholesterol, BMI

**^b^R^2^ = _0.029_ (Adjusted R^2^ = _0.020_),** dependent variable: hand grip, independent covariate: age, CoQ_10_/cholesterol, BMI

BMI = body mass index; df = degrees of freedom, *F* = variance ratio

Thus, we intended to test our hypothesis in an independent validation study population, which was characterized, on average, by a higher number of subjects, higher mean age and higher mean BMI values. When we analyzed the influence of the variables that predict the outcome of upper body muscular strength (hand grip, dependent factor) in ANCOVA with gender as an independent factor and CoQ_10_/cholesterol and BMI as independent covariates, gender was found to be the strongest effector of hand grip (p<0.001, η^2^ = 0.601, data not shown). However, when we analyzed the remaining variables independent of gender, we found a significant effect on hand grip by CoQ_10_/cholesterol (p<0.001, η^2^ = 0.019) and age (p<0.001, η^2^ = 0.018), whereas in this study population, which is characterized by a higher percentage of obese subjects, BMI was no longer a significant predictor of hand grip (p = 0.896, Table 3B).

## Discussion

Aging is associated with sarcopenia, which is a loss of skeletal muscle mass and function [35]. CoQ_10_ plays a crucial role in mitochondrial bioenergetics and can act as an antioxidant [7,36]. However, CoQ_10_ as a determinant of muscular strength is not well documented. Therefore, this study is, to the best of our knowledge, the first to examine the determinants of muscular strength with a special focus on the serum CoQ_10_ status in two independent cohorts. We showed that the CoQ_10_/cholesterol ratio was significantly correlated with a lower hand grip and a predictor of hand grip outcome. Gender was found to be the main influencing factor. Age was also significantly negatively correlated with muscle strength, but only in older subjects (males and females) of the validation population, which exhibited a mean age of 52.6 ± 14.1 years in males or 39.8 ± 11.5 years in females, of the basic study population. Interestingly, the positive association between hand grip and BMI was dependent on the BMI classes because we found a significant correlation in the subjects of the validation population who exhibited a normal BMI, but not in overweight or obese subjects (Fig 2B). This finding was irrespective of gender. We conclude that in a normal BMI range, an increase in BMI equals an increase in muscle mass, whereas in obese subjects (BMI>30), an increase in BMI possibly equals an increase in fat mass and hence does not necessarily lead to an increase in muscle strength. In our basic study population, which had a mean BMI of 26.0 ± 4.4, BMI was significantly correlated with hand grip strength (Fig 1B) and also with muscle mass (r = 0.205, p<0.001), which agrees with our hypothesis. Similarly, in a population of elderly women, higher BMI values were associated with a greater probability of a functional limitation [37]. Here, the isometric leg strength was significantly lower in subjects with sarcopenia and sarcopenic obesity. In an observational cross-sectional study, older men and women with weak muscle strength and higher BMI had considerably poorer performance than others [38]. The authors suggested a very likely benefit of early assessment and interventions to reduce fat mass and improve muscle strength in the prevention of future functional limitations.

The relationship of obesity and health and CoQ_10_ as a major lipophilic antioxidant and mitochondrial respiratory chain redox coupler [39] is currently a matter of debate. Recently, the impact of aging, BMI and physical capacity on the CoQ_10_ levels in human blood was investigated [8]. In a cohort of community-dwelling people (n = 43, mean age = 71.01 ± 6.22 years), the impact of hand grip measurements and further parameters of physical activity on the CoQ_10_ plasma level was studied. The authors found that people who exhibited higher levels of functional capacity presented lower levels of cholesterol, which was accompanied by higher levels of CoQ_10_ in plasma. On the other hand, obesity was related to lower CoQ_10_ levels. They concluded that physical activity at advanced ages can increase the levels of CoQ_10_ and reduce the levels of lipid peroxidation in plasma, probably reducing the progression of cardiovascular diseases. In accordance with these findings, in our validation population, cholesterol itself as well as the CoQ_10_ redox state were significantly negative correlated with hand grip. For the observed lower level of CoQ_10_/cholesterol in the validation population, we cannot fully rule out that it could be partly caused by normalization to the significantly different cholesterol level. Hence, we showed that in this older and obese study group, the subjects who exhibited a lower level of muscular strength presented a higher level of cholesterol and a lower percentage of reduced CoQ_10_ (ubiquinol). In this context, it is worthwhile to mention that physical activity has been shown to generate reactive oxygen species (ROS) [40]; however, on the other hand, it has also been demonstrated to be a preventive mechanism against oxidative stress. Different training degrees have been suggested to be beneficial in humans by enhancing the antioxidant capacity [41]. Importantly, it has also been shown by Ristow and coworkers [42] that dietary antioxidants could counteract the health-promoting effects of physical exercise due to a suppression of endogenous antioxidant defense mechanisms (the so-called “hormesis” effect). In addition to upper body muscular strength (hand grip), we also indicated that peak expiratory flow (PEF) is a function of respiratory muscle, which could also be affected in age-associated alterations in skeletal muscles, which has been shown to be correlated with hand grip strength [24]. In a study comprising data from 960 older individuals, pulmonary function was suggested to partially account for the association of muscle strength and mortality [43]. In the present study, PEF was also significantly correlated with hand grip strength in the basic study population. Furthermore, when we correlated CoQ_10_/cholesterol versus PEF, a significant Spearman correlation coefficient (r = 0,138, p<0.05) was evident (data not shown). Similarly, CoQ_10_/cholesterol was significantly correlated with muscle mass (r = 0.1425, p<0.01).

CoQ_10_ supplementation increases plasma levels of CoQ_10_ [44–46]. Nevertheless, short-term dosing of CoQ_10_ in middle-aged, untrained men did not improve the aerobic capacity of forearm exercise metabolism [47]. On the other hand, a placebo controlled study with supplementation of 300 mg of ubiquinol for 6 weeks significantly enhanced physical performance, which was measured as the maximum power output in young healthy trained Olympic athletes [48]. A systematic review to analyze the influence and effect of CoQ_10_ supplementation on parameters related to exercise in healthy humans revealed that CoQ_10_ has the potential to be used as a nutritional supplement to improve exercise capacity; however, the current literature shows a disparity and inconsistency, possibly due to differences in the CoQ_10_ formulations used, dosage, timing of the supplement, exercise tests performed and study participants [49].

In conclusion, the present data suggest that gender and CoQ_10_/cholesterol are determinants of muscular strength, with gender being the main influencing factor. In older subjects, age is an additional inverse determinant of muscular strength, whereas the body mass of a person ascertains muscular strength only when in the normal range. A lower CoQ_10_/cholesterol level could be a predictor of an increased risk of sarcopenia in humans due to its association to upper body muscle strength, peak flow and muscle mass. The present study provides further evidence that a higher muscular strength or physical activity in risk groups, including older and obese subjects, could lead to a more a favored outcome in the cholesterol metabolism, CoQ_10_ and CoQ_10_ redox status.

## Supporting Information

S1 Table Raw data of the A) Basic study population and the B) Validation population.
(XLSX)
